# Improved Natamycin Production in *Streptomyces gilvosporeus* Through Mutagenesis and Enhanced Nitrogen Metabolism

**DOI:** 10.3390/microorganisms13020390

**Published:** 2025-02-10

**Authors:** Liang Wang, Wen Xiao, Hongjian Zhang, Jianhua Zhang, Xusheng Chen

**Affiliations:** Key Laboratory of Industrial Biotechnology, Ministry of Education, School of Biotechnology, Jiangnan University, Wuxi 214122, China; wangl@jiangnan.edu.cn (L.W.);

**Keywords:** natamycin, *Streptomyces gilvosporeus*, ARTP mutagenesis, 2-deoxyglucose, nitrogen metabolism regulation

## Abstract

Natamycin is a polyene macrocyclic antibiotic extensively used in food, medical, and agricultural industries. However, its high production cost and low synthetic efficiency fail to meet the growing market demand. Therefore, enhancing the production of natamycin-producing strains is crucial for achieving its industrial-scale production. This study systematically evaluated 16 mutagenesis methods and identified atmospheric and room temperature plasma mutagenesis combined with 2-deoxyglucose tolerance screening as the optimal strategy for enhancing natamycin production. A high-yield mutant strain, AG-2, was obtained, achieving an 80% increase in natamycin production (1.53 g/L) compared to the original strain. Metabolic analysis revealed that glycolysis and the pentose phosphate pathway were enhanced in AG-2, while the tricarboxylic acid cycle was weakened, significantly increasing the supply of precursors such as acetyl-CoA, methylmalonyl-CoA, and the reducing power of NADPH. Additionally, overexpression of the nitrogen metabolism regulatory gene *glnR* promoted the supply of glutamate and glutamine, further increasing natamycin production in AG-2 to 1.85 g/L. In a 5 L fermenter, the engineered strain AG-*glnR* achieved a final natamycin production of 11.50 g/L, 1.67 times higher than the original strain. This study is the first to combine mutagenesis with nitrogen metabolism regulation, effectively enhancing natamycin production and providing a novel approach for the efficient synthesis of other polyene antibiotics.

## 1. Introduction

**Figure 1:**
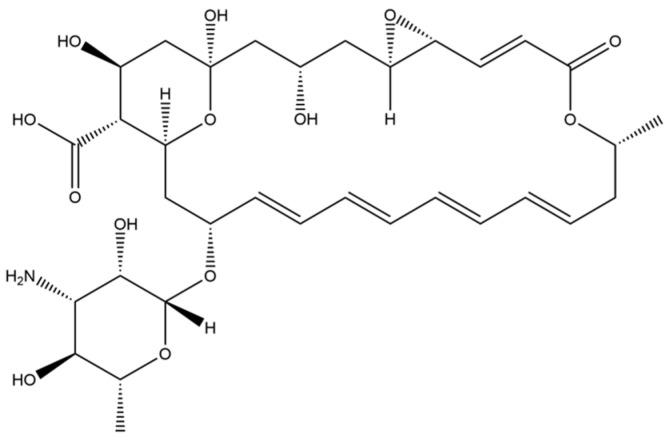
The chemical structure of natamycin.

Natamycin is a polyene macrocyclic antibiotic mainly produced by *Streptomyces natalensis*, *Streptomyces chatanoogensis*, *Streptomyces lydicus*, and *Streptomyces gilvosporeus* (Figure 1) [1]. This antibiotic can effectively inhibit yeasts and molds, thereby preventing the production of fungal toxins [2]. Due to its low toxicity, high safety, stability, and lack of side effects on mammalian cells, natamycin is widely used in the food industry [3]. Currently, natamycin has been approved as a food preservative in over 50 countries worldwide [4]. It is also extensively used in the fields of medicine and agriculture [5]. However, high production costs and low synthetic efficiencies are still the main factors limiting the application of natamycin. Therefore, enhancing the production of natamycin-producing strains is crucial for achieving industrial-scale production [6].

Genetic engineering is a powerful tool to increase natamycin production. For example, Wu et al. successfully overexpressed *slnM*, a positive regulatory gene for natamycin production in *S. lydicus*. This resulted in the acquisition of a strain with a production of 2.50 g/L, exhibiting a 22% increase compared to the parental strain [7]. Jiang et al. conducted genetic modification on the phosphoribosyltransferase gene and obtained a natamycin high-yield mutant sHJ003. The natamycin production of sHJ003 reached 3.12 g/L, representing a 40% increase over the parental strain *Streptomyces chattanoogensis* L10 [8]. Although metabolic engineering is effective in enhancing the fermentation performance of strains, identifying key pathways and genetic elements remains challenging due to the complex microbial genetic background. Consequently, traditional mutagenesis methods continue to be regarded as effective approaches for improving production efficiency. Due to differences in their mechanisms of action, mutagenesis strategies exhibit distinct effects on the same strain (e.g., positive mutation rates) [9]. For instance, ultraviolet (UV) mutagenesis induces the formation of DNA pyrimidine dimers, resulting in replication errors and subsequent point mutations. Nitrosoguanidine (NTG) mutagenesis causes DNA depurination or depyrimidination via nitrosation, resulting in base mispairing. Diethyl sulfate (DES) mutagenesis introduces ethyl groups into DNA through alkylation reactions, causing base substitutions [10,11,12]. Therefore, selecting an appropriate mutagenesis strategy for the specific strain is crucial for effective strain improvement.

Additionally, a combination of mutagenesis and tolerance screening is often utilized to improve the positive mutation rate and strain improvement efficiency. Many studies indicate that increasing the tolerance of certain target traits promotes the production of secondary metabolites [13,14]. 2-deoxyglucose (2-DG) is a structural analog of glucose. Screening for strains tolerant to 2-DG effectively alleviates the glucose repression effect, while simultaneously promoting the synthesis of secondary metabolites [15,16]. Streptomycin can bind to ribosomal proteins, thereby inhibiting the translation process and leading to strain death [17]. Mutations conferring streptomycin resistance lead to a significant enhancement in secondary metabolite production [18,19]. Yu et al. used genome shuffling and ribosome engineering to obtain a high-daptomycin-producing *Streptomyces roseosporus* PRGN21 mutant, which produced 324 mg/L of daptomycin, nearly four times higher than the parental strain [20]. Lithium chloride, a co-mutagenic metal, enhances the mutagenic effects of mutagens like UV in the development of antibiotic-producing strains. Song et al. used UV with lithium chloride to screen for high avermectin B1a producers, resulting in a genetically stable *Streptomyces avermitilis* S-233 strain with a production of 6818 µg/mL, 23.8% higher than the parental strain [21]. Natamycin biosynthesis is inhibited when phosphate levels exceed 1 mmol/L, though phosphate is essential for strain growth. Marta et al. increased natamycin production by 80% by knocking down the PhoP/R regulatory system, reducing the strain’s phosphate sensitivity [22]. In conclusion, increasing the adaptability of natamycin-producing strains is an effective method for the rapid increase in natamycin production.

To enhance natamycin production, sixteen mutagenesis-based strategies were systematically evaluated to identify the most effective approach for improving both the positive mutation rate and natamycin production in *S. gilvosporeus*. The results showed that combining atmospheric and room temperature plasma (ARTP) mutagenesis with 2-DG tolerance screening was the most efficient strategy. After two rounds of screening, the mutant strain AG-2 was obtained, exhibiting the highest natamycin production among all mutants. Further gene expression and metabolic analysis provided insights into the high-yield mechanism of AG-2. Finally, optimization of nitrogen metabolism further increased natamycin production. This study lays a theoretical foundation for the development of advanced *Streptomyces* cell factories for high-value product production.

## 2. Materials and Methods

### 2.1. Strains and Culture Conditions

*S. gilvosporeus* ATCC 13326 and its derivatives were cultured on Mannitol Soybean (MS) agar plates, a commonly used medium for *Streptomyces* sporulation. For shake flask fermentation, 100 μL of spore suspension was inoculated into a 250 mL flask containing 30 mL of fresh medium composed of 20 g/L yeast extract, 50 g/L dextrose, 10 g/L ammonium sulfate, 1.36 g/L potassium dihydrogen phosphate, 0.8 g/L dipotassium hydrogen phosphate, 0.5 g/L magnesium sulfate, 0.04 g/L zinc sulfate, and 0.03 g/L ferrous sulfate, with the pH adjusted to 7.0. The flask was incubated for 24 h to serve as the seed culture. Subsequently, 1.8 mL of the seed culture was transferred into 30 mL of a fermentation medium, which contained 60 g/L glucose, 20 g/L soybean peptone, 4.5 g/L yeast extract, 1 g/L magnesium sulfate, and 2 g/L sodium chloride, and the pH was adjusted to 7.0. The fermentation medium was incubated for 7 d at 28 °C with shaking at 220 rpm in a rotary shaker.

### 2.2. Agar Diffusion Method

The natamycin production capacity of different strains was evaluated using the agar diffusion method. Mycelium-overgrown MS plates were punched with an 8 mm hole punch to obtain agar columns, which were then incubated in a humidified chamber for two days to allow natamycin diffusion. Subsequently, double-layer bioassay plates were prepared by adding 10^7^ CFU/mL of *Saccharomyces cerevisiae* as the sensitive indicator strain to the upper layer of the Yeast Extract Peptone Dextrose medium. After placing the agar column on the bioassay plate, the plates were incubated at 28 °C for 12 h. The diameter of the inhibition zone correlates with natamycin production.

### 2.3. Minimum Inhibitory Concentration

Spore suspensions were prepared with varying concentrations of streptomycin (0.2, 0.5, 1.0, 2.0, 4.0, 6.0, 8.0, 10.0, 12.0 μg/mL), 2-deoxyglucose (0.5, 1.0, 2.0, 3.0, 5.0, 7.0, 10.0 g/L), and lithium chloride (0.5, 1.0, 3.0, 5.0, 10.0 g/L). Additionally, sodium chloride (1.0, 2.0, 3.0, 5.0, 7.0, 10.0 g/L), lithium chloride (0.025%, 0.05%, 0.1%, 0.2%, 0.4%, 0.8%, 1.5%), and potassium phosphate (0.025, 0.05, 0.1, 0.2 g/L) concentrations were also prepared. Colony growth was monitored, and the minimum concentration at which no growth occurred was recorded as the minimum inhibitory concentration.

### 2.4. Mutagenesis Methods

UV mutagenesis was performed using a 15 W UV lamp at a distance of 20 cm, irradiating the spore suspension for 5, 10, 15, 20, 25, 30, 40, and 60 s. For ARTP mutagenesis, 10 μL of spore suspension was applied to mutagenized slides at an operating voltage of 100 W, an irradiation distance of 2 mm, and an airflow rate of 10 slpm (standard liters per minute), with exposure times of 0, 40, 60, 80, 100, and 120 s. DES mutagenesis was carried out by adding 1 mL of spore suspension to 9 mL of PBS buffer and 0.1 mL of NTG solution at 28 °C for 0, 10, 20, 30, 40, and 60 min. The reaction was terminated by adding 25% sodium thiosulfate. A total of 100 μL of the mutagenized spore suspension was then plated on MS agar plates and incubated at 28 °C for 3–4 d. For NTG mutagenesis, 0.1 mL of NTG was added to 10 mL of bacterial suspension to achieve a final concentration of 1%. Samples (1 mL) were taken at 0, 10, 20, 30, 40, and 60 min and immediately diluted 100-fold, and 100 μL of the diluted mutagenized culture was spread onto MS agar plates. The plates were incubated at 28 °C until single colonies appeared.

### 2.5. Field Emission Scanning Electron Microscopy Analysis

Mycelia of *S. gilvosporeus* ATCC 13326 and the high-yield mutant strain AG-2, treated with or without 1% (*v*/*v*) ethanol, were collected and washed twice with PBS. The mycelia were then fixed with 3% (*v*/*v*) glutaraldehyde for 2 h at 4 °C. After fixation, the mycelia were washed, centrifuged, and dehydrated through a gradient ethanol series. Mycelial morphology was examined using field emission scanning electron microscopy.

### 2.6. RNA Extraction and Quantitative Real-Time PCR (qRT-PCR)

Total RNA was extracted using the Trizol reagent (Shanghai Sangong Biotechnology Co., Ltd., Shanghai, China). cDNA synthesis was performed using the IiSint III RT SuperMix for the qPCR (+gDNAwiper) kit (Vazyme Biotech Co., Ltd., Nanjing, China), following the manufacturer’s instructions. Gene-specific primers for the target gene were designed with Beacon Designer 7 software. qPCR was performed on the StepOne Real-Time PCR system (Applied Biosystems, Foster City, CA, USA) using SYBR^®^ Premix Ex Taq™ (Takara, Kyoto, Japan). The PCR protocol included an initial denaturation at 95 °C for 30 s, followed by 40 cycles of denaturation at 95 °C for 10 s and extension at 60 °C for 30 s. Each reaction was carried out in a 20 μL volume containing 10 μL of 2 × ChamQ Universal SYBR qPCR Master Mix, 2 μL of DNA/cDNA template, 0.4 μL of forward and reverse primers (10 μM), and 7.2 μL of ddH_2_O. Relative quantification of the target gene was performed using the 2^−∆∆Ct^ method, and 16S rRNA was used as the internal reference gene. The transcript level of the target gene in the control was set to 1, and results are presented as fold change relative to the control. Primer sequences for the relevant genes are listed in Table S1. The gene sequences used in this study were derived from the genome of *Streptomyces gilvosporeus* F607 (GenBank: GCA_002082195.1), which serves as the reference genome.

### 2.7. Analysis Methods

The growth of *S. gilvosporeus* ATCC 13326 and *S. gilvosporeus* AG-2 was evaluated by the dry cell weight (DCW) method. A 4 mL sample of fermentation broth was centrifuged at 11,400× *g* for 10 min. The resulting precipitate was dried at 105 °C for 12 h and then weighed. Residual glucose was measured using a biosensor (Centrifuge 5804 R, Institute of Biology, Shandong Academy of Sciences, Jinan, China). Natamycin production was determined by high-performance liquid chromatography. For this, 1 mL of fermentation broth was mixed with 9 mL of 5% glacial acetic acid in methanol. The mixture was then centrifuged at 2960× *g* for 25 min, and the supernatant was filtered through a 1 mL needle and an organic membrane. The separation was performed on a C18 reversed-phase column (10 μL, 250 × 4.6 mm) using a mobile phase of methanol/water = 65:35. The detection wavelength was 303 nm, with a flow rate of 1 mL/min and an injection volume of 10 μL.

### 2.8. Statistical Analysis

All experiments were conducted in triplicate, and data are presented as mean ± standard deviation. Statistical analysis was performed using SPSS (version 22.0, SPSS Inc., Chicago, IL, USA), with a one-way analysis of variance (ANOVA) followed by Tukey’s test (*p* < 0.05) for comparisons.

## 3. Results

### 3.1. The Optimal Strategy for Screening Natamycin High-Yield Mutants

To identify the optimal strategy, we separately applied ARTP, UV, DES, and NTG mutagenesis to enhance the strains’ tolerance to streptomycin, 2-DG, KH_2_POy the optimal strategy, we separately, and LiCl. The optimal mutagenesis treatment time and the appropriate concentrations of streptomycin, 2-DG, KH_2_PO_4_, and LiCl were critical factors in achieving a high mutation rate. We assessed the lethality of the strains under each mutagenesis condition, and the resulting lethality rate curves are shown in Figure S1. Additionally, the minimum inhibitory concentrations of streptomycin, 2-DG, KH_2_PO_4_, and LiCl were determined, and the results are presented in Figure S2.

**Figure 2:**
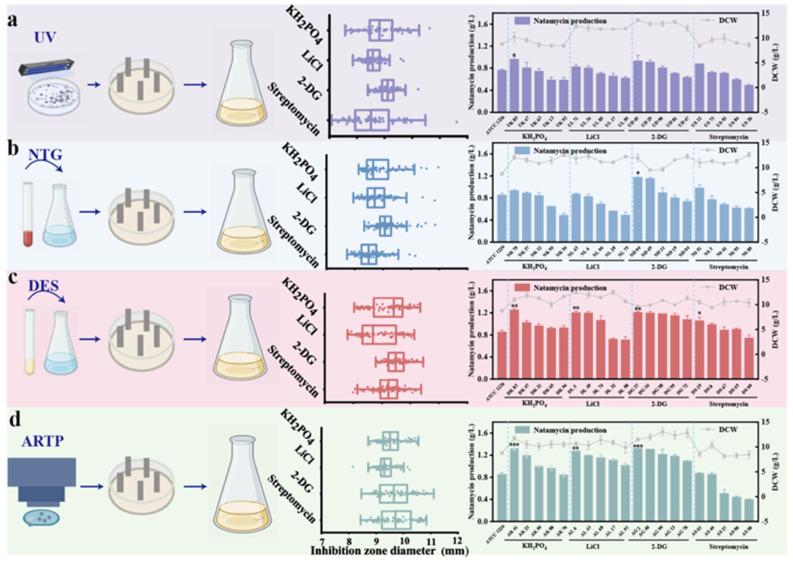
Different strategies for screening natamycin high-producing mutants. (**a**) Strategies based on UV mutagenesis; (**b**) strategies based on NTG mutagenesis; (**c**) strategies based on DES mutagenesis; and (**d**) strategies based on ARTP mutagenesis. In each method, the strains were mutagenized and then coated onto plates containing varying concentrations of streptomycin, 2-DG, KH_2_PO_4_, and LiCl. The plates were then incubated at 28 °C for 8 d. Natamycin production and dry cell weight (DCW) of mutants were measured after 96 h of shake flask fermentation. Each fermentation experiment was performed in triplicate, and the results were expressed as the mean of three independent replicates, with error bars representing the standard deviation. * indicates significance at *p* < 0.05, ** indicates significance at *p* < 0.01, and *** indicates significance at *p* < 0.001.

Fresh spore suspensions of strain *S. gilvosporeus* ATCC 13326 were treated with ARTP, UV, DES, and NTG, and the mutagenized strains were then spread on MS plates containing lethal concentrations of streptomycin, 2-DG, KH_2_PO_4,_ and LiCl, respectively. After 6 d of incubation, 100 mutants from each mutagenesis strategy were screened using the agar diffusion method (Figure 2a–d). The top five strains with the largest inhibition zones in each strategy were selected for shake flask fermentation. The positive mutation rate and the highest natamycin production from the 16 strategies in shake flask fermentation are shown in Table 1. The results revealed that the ARTP combined with the 2-DG tolerance mutagenesis strategy achieved the highest positive mutation rate (54.12%). Therefore, this strategy was used as the optimal mutagenesis approach and applied in the subsequent high-yield strain development process.

### 3.2. Screening for High-Natamycin-Producing Mutants via ARTP Mutagenesis Combined with 2-DG Tolerance

**Figure 3:**
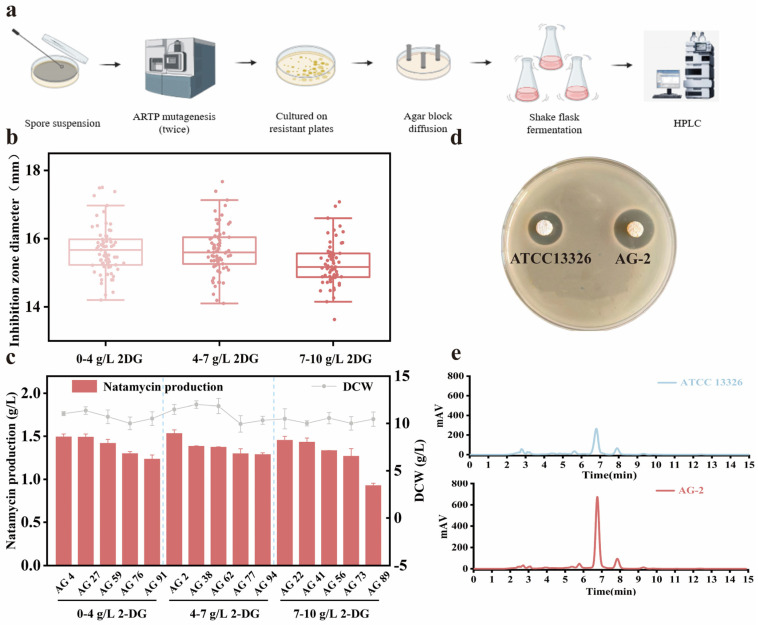
Screening for natamycin-producing strains using ARTP mutagenesis combined with 2-DG. (**a**) Workflow of ARTP mutagenesis combined with 2-DG tolerance screening; (**b**) agar diffusion method to assess the antimicrobial activity of the mutants; (**c**) natamycin production and dry cell weight (DCW) of the five highest-yielding mutants obtained on plates with different phosphate concentrations; (**d**) comparison of the inhibition zone diameter between *S. gilvosporeus* ATCC 13326 and *S. gilvosporeus* AG-2; and (**e**) HPLC analysis of natamycin production in *S. gilvosporeus* ATCC 13326 and *S. gilvosporeus* AG-2. Fermentation was repeated three times. Data are presented as the mean of three independent cultures, with error bars representing standard deviation.

To further investigate the effect of varying phosphate concentrations on natamycin production, mutant AG-1 was selected as the starting strain for the second round of ARTP mutagenesis (Figure 3a). After ARTP mutagenesis, the resulting mutants were evenly divided into three groups and plated onto MS plates with phosphate concentration gradients of low (0–4 g/L), medium (4–7 g/L), and high (7–10 g/L). One hundred mutants were selected from each concentration range. Natamycin production was evaluated using the agar diffusion method, and the five mutants with the largest inhibition zone diameters were chosen for shake flask fermentation (Figure 3b,c). The results showed positive mutation rates of 8%, 10%, and 4% for strains on plates containing 2-DG concentrations of 0–4 g/L, 4–7 g/L, and 7–10 g/L, respectively. The highest natamycin production (1.53 g/L) was observed under the 4–7 g/L 2-DG condition, surpassing the productions of 1.49 g/L and 1.45 g/L at the 0–4 g/L and 7–10 g/L concentrations, respectively. Ultimately, the mutant strain AG-2 was obtained, with natamycin production and DCW reaching 1.53 g/L and 10.95 g/L, respectively, representing increases of 80% and 25.86% compared to ATCC 13326 (Figure 3d,e).

### 3.3. Changes in Mycelial Morphology and Fermentation Performance

**Figure 4:**
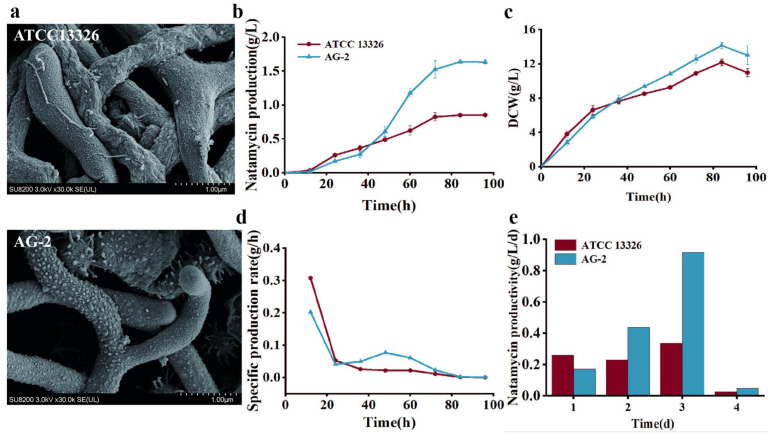
Physiological differences between *S. gilvosporeus* ATCC 13326 and *S. gilvosporeus* AG-2. (**a**) Mycelial morphology; (**b**) natamycin production; (**c**) dry cell weight (DCW); (**d**) specific production rate; and (**e**) productivity.

Mutagenesis often induces macroscopic or microscopic morphological changes in microorganisms. Therefore, field emission scanning electron microscopy was used to observe the mycelial and spore morphology of ATCC 13326 and mutant AG-2. However, the high-yield strain AG-2 showed no significant morphological differences compared to the wild-type ATCC 13326 (Figure 4a). This result suggests that the mutations may be primarily focused on enhancing metabolic pathways without affecting the phenotype of the strain AG-2.

### 3.4. Changes in Fermentation Performance

The fermentation performance of high-yield mutant AG-2 and the original strain was evaluated in shake flasks. As shown in Figure 4b, natamycin production for both strains increased rapidly before 72 h and then plateaued. Before 36 h, ATCC 13326 produced significantly more natamycin than AG-2, but after 36 h, the situation reversed. Natamycin production of AG-2 reached 1.53 g/L at 72 h, 80% higher than that of ATCC 13326. The DCW of AG-2 was lower than the control before 48 h, but surpassed it thereafter, peaking at 84 h (Figure 4c). Additionally, the specific production rate of AG-2 was consistently higher than that of ATCC 13326 from 24 to 72 h (Figure 4d). The productivity of both strains peaked on the third day and dropped to zero on the fifth day (Figure 4e). AG-2 exhibited higher productivity than the parental strain on all days except the first day of fermentation.

### 3.5. Transcriptional Changes in Key Enzymes of Carbohydrate Metabolism

**Figure 5:**
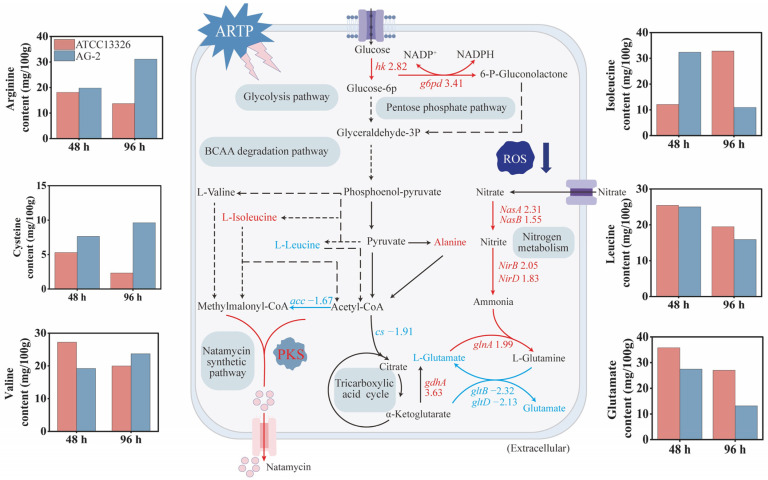
Key genes and metabolic changes in the natamycin biosynthetic pathway in the natamycin high-producing strain AG-2. Red indicates upregulated genes/metabolites, while blue indicates downregulated genes/metabolites. The red numbers represent the fold changes in upregulated genes, and the blue numbers represent the fold changes in downregulated genes.

To investigate the physiological mechanisms behind the increased natamycin production in AG-2, we compared the transcriptional levels of key enzymes involved in these metabolic pathways between AG-2 and the parental strain. In AG-2, the transcriptional level of hexokinase (encoded by *hk*) in glycolysis was 2.82 times higher than that in the parental strain (Figure 5). Similarly, glucose-6-phosphate dehydrogenase (encoded by *g6pd*) in the PP pathway showed a 3.41-fold increase, while citrate synthase (encoded by *cs*) in the TCA cycle decreased by 47.6% (Figure 5).

### 3.6. Transcriptional Differences in Key Genes Involved in the Natamycin Biosynthesis Clusters

The transcriptional levels of key genes involved in natamycin biosynthesis were analyzed by qRT-PCR (Table S2). The results showed that 17 out of 19 genes in the natamycin biosynthetic gene cluster were upregulated in the high-yield strain AG-2. Specifically, the transcript levels of genes encoding polyketide synthase, including *sgnS0*, *sgnS1*, *sgnS2*, *sgnS3*, and *sgnS4*, were significantly upregulated by 8.45-fold, 3.71-fold, 5.83-fold, 4.64-fold, and 7.37-fold, respectively. Genes involved in modifying the natamycin backbone, such as *sgnK*, *sgnI*, *sgnJ*, *sgnC*, *sgnG*, *sgnF*, and *sgnD*, exhibited upregulation by 23.92-fold, 16.12-fold, 26.71-fold, 19.43-fold, 27.86-fold, 11.89-fold, and 19.53-fold, respectively. Additionally, genes encoding natamycin transporter proteins, including *sgnA*, *sgnB*, and *sgnH*, were upregulated by 13.76-fold, 28.81-fold, and 2.45-fold, respectively. Regulatory genes for natamycin transport, *sgnE* and *sgnM*, showed upregulation by 7.71-fold and 9.30-fold, respectively. However, the transcription levels of the regulatory gene *sgnR* and the structural gene *sgnL* were slightly downregulated by 1.22-fold and 1.65-fold, respectively.

### 3.7. Differences in Intracellular Amino Acid Levels

As shown in Figure 5, the intracellular levels of arginine and cysteine in AG-2 were consistently higher than those in the original strain at both 48 h and 96 h. During the early fermentation phase, the intracellular isoleucine level in AG-2 was 2.67 times that of the original strain, while the valine level was 29.4% lower. Valine supplementation experiments revealed that adding 0.50 g/L of valine at 24 h increased natamycin production by 8.43% (Figure S3). Additionally, the intracellular glutamate level in AG-2 was 23.4% lower than that in the original strain. Glutamate is primarily synthesized through two pathways: (1) the reductive amination of α-ketoglutarate by glutamate dehydrogenase (encoded by *gdhA*) and (2) the reaction of glutamine with α-ketoglutarate, catalyzed by glutamate synthase (GOGAT, encoded by *gltB* and *gltD*), with the consumption of NADPH to produce glutamate (Figure 5). To explore the cause of the reduced intracellular glutamate, we analyzed the transcriptional changes in key genes in these biosynthesis pathways. In AG-2, the transcriptional level of *gdhA* was upregulated by 3.63-fold, suggesting enhanced conversion of α-ketoglutarate to glutamate. Additionally, the transcription levels of genes involved in nitrogen assimilation, including *nasB*, *nasA*, *nirB*, *nirD*, and *glnA*, were upregulated, promoting the synthesis of glutamine and glutamate (Figure 5).

In conclusion, the increased intracellular levels of arginine, cysteine, and isoleucine may contribute to the high natamycin production in AG-2. However, the glutamate supply appears to be insufficient, and enhancing the availability of glutamate and glutamine may further improve natamycin production.

### 3.8. Enhancing Nitrogen Metabolism for Natamycin Production

**Figure 6:**
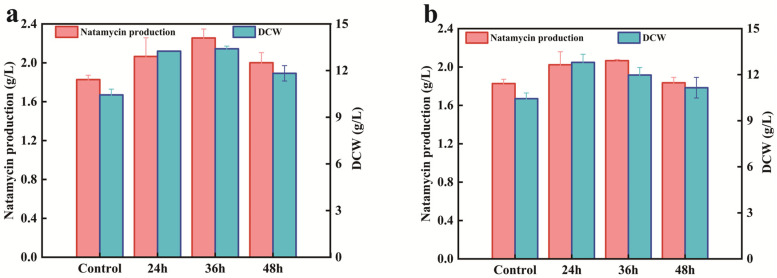
Effect of nitrogen metabolism on natamycin production by *S. gilvosporeus* AG2. (**a**) Exogenous addition of glutamate at 24, 36, and 48 h on natamycin production of AG-2; (**b**) exogenous addition of glutamine at 24, 36, and 48 h on natamycin production of AG-2. DCW: dry cell weight.

Amino acid feeding experiments showed that the addition of glutamate and glutamine at a final concentration of 0.32 g/L at 24 h and 36 h, respectively, significantly increased natamycin production in AG-2 (Figure 6a,b). This finding highlights the promoting effect of glutamate and glutamine on natamycin synthesis. Since glutamate and glutamine are key products of nitrogen metabolism, further enhancement of nitrogen metabolic pathways in AG-2 may significantly promote natamycin production. GlnR, a key regulator of nitrogen metabolism, controls multiple nitrogen metabolism-related genes simultaneously [23,24]. Therefore, we hypothesize that GlnR might play a role in natamycin synthesis.

**Figure 7:**
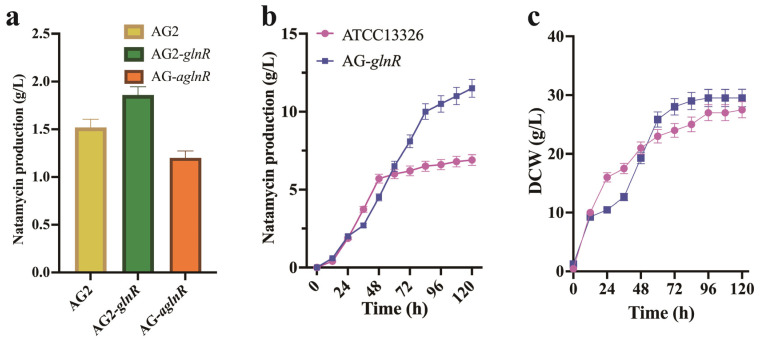
Enhancement of nitrogen metabolism increases natamycin production in AG2. (**a**) Effect of GlnR overexpression and inhibition on natamycin production. (**b**) Comparison of natamycin production between *S. gilvosporeus* AG2-*glnR* and the parental strain *S. gilvosporeus* ATCC 13326 in 5 L bioreactors. (**c**) Comparison of dry cell weight (DCW) between *S. gilvosporeus* AG2-*glnR* and the parental strain *S. gilvosporeus* ATCC 13326 in 5 L bioreactors.

To assess the impact of GlnR on natamycin production, the *glnR* gene was first amplified from *S. gilvosporeus* ATCC13326. It was then inserted into the pIB139 vector under the control of the ermEp promoter to create the overexpression plasmid pIB139-*glnR*. This expression vector was subsequently introduced into *S. gilvosporeus* via conjugation, resulting in the engineered strain AG-*glnR*. Shake flask fermentation results showed that AG-*glnR* produced 1.85 g/L of natamycin after 96 h of fermentation, representing a 20.9% increase compared to AG-2 (Figure 7a). Subsequently, the AG-*aglnR* strain was also constructed, in which the translation of the *glnR* gene was inhibited using antisense RNA (Figure 7a). The natamycin production of AG-*aglnR* was 1.20 g/L, 20.52% lower than AG-2. These results suggest that GlnR positively regulates nitrogen metabolism, thereby enhancing natamycin synthesis in AG-2.

Finally, we evaluated the fed-batch fermentation performance of the high-yield strain AG-*glnR* in a 5 L fermenter. As fermentation progressed, the DCW of AG-*glnR* increased rapidly to 21.32 g/L by 48 h, and the growth rate slowed after 72 h. Natamycin production began to increase from 36 h, reaching 11.51 g/L after 120 h of fermentation, 67% higher than that of ATCC 13326 (Figure 7b,c). The DCW of AG-*glnR* (29.51 g/L) was comparable to that of ATCC 13326 (27.32 g/L).

## 4. Discussion

Natamycin, a natural preservative and antifungal agent, is of great industrial importance due to its potential for large-scale production. However, the complexity of its biosynthetic pathway and unclear regulatory mechanisms have hindered efforts to improve its production. In this study, the natamycin production capacity of *S. gilvosporeus* was significantly enhanced by optimizing mutagenesis strategies and improving nitrogen metabolism. Among 16 mutagenesis methods tested, ARTP mutagenesis combined with 2-DG tolerance screening was identified as the most effective approach for improving natamycin production. A high-yield mutant strain, AG-2, was obtained, producing 1.53 g/L of natamycin, an 80% increase compared to the original strain. Further analysis revealed that the coordinated regulation of carbohydrate and nitrogen metabolism pathways played a key role in the high-yield production of AG-2. Further enhancing nitrogen metabolism in AG-2 resulted in a 20.9% increase in natamycin production.

Our study found that ARTP mutagenesis combined with 2-DG tolerance screening significantly enhanced the natamycin production of *S. gilvosporeus*. ARTP mutagenesis is an efficient method that uses plasma jets to disrupt cell membrane structures, inducing genetic damage and mutations [25,26]. Compared to other mutagenesis techniques, ARTP mutagenesis is capable of inducing mutations in multiple genes simultaneously, allowing for faster and more efficient gene modification [27,28]. This makes ARTP particularly suitable for the rapid selection of 2-DG-tolerant mutant strains. We also found that enhancing the strain’s tolerance to 2-DG significantly increased natamycin synthesis. This improvement is likely due to the fact that the original strain, ATCC 13326, is sensitive to the byproducts of glucose metabolism, and 2-DG tolerance alleviated metabolic repression, thereby promoting cell growth and natamycin production. Moreover, it was observed that mutant strains obtained from plates containing lower concentrations of 2-DG exhibited higher natamycin production (Figure 3c). This result is consistent with findings from Zhu et al. [29], who observed that low gentamicin concentrations led to a higher frequency of increased antibiotic production in mutant strains (3–20%). This suggests that low-concentration resistance screening may be more effective for enhancing secondary metabolite production in *Streptomyces* and other microorganisms.

Carbohydrate metabolism plays a crucial role in natamycin biosynthesis. Acetyl-CoA and malonyl-CoA are key precursors for natamycin biosynthesis, regulated by the glycolysis pathway, pentose phosphate (PP) pathway, and tricarboxylic acid (TCA) cycle [30,31,32]. Compared to the original strain, AG-2 exhibited enhanced glycolysis and PP pathways, while the TCA cycle was downregulated. The upregulation of glycolysis and the PP pathways increased the supply of pyruvate and NADPH, which are essential for the synthesis of acetyl-CoA and malonyl-CoA, as well as for providing reducing power during natamycin biosynthesis [33]. Meanwhile, the downregulation of the TCA cycle reduced acetyl-CoA consumption, leading to its intracellular accumulation. This coordinated regulation of metabolic pathways likely contributes to the significantly enhanced natamycin production in AG-2. Supporting this, Wang et al. found that reduced citrate synthase activity, combined with increased phosphoenolpyruvate carboxylase and pyruvate carboxylase activities, can effectively increase acetyl-CoA levels, thereby improving natamycin biosynthesis efficiency [34]. These findings further validate the role of carbohydrate metabolism regulation in enhancing the accumulation of natamycin precursors.

Intracellular amino acids play a critical role in the synthesis of secondary metabolites by modulating the supply of precursors. Cysteine, a precursor of glutathione, possesses strong antioxidant properties. The accumulation of cysteine in AG-2 may alleviate oxidative stress-induced damage and enhance the cell’s antioxidant capacity, thereby providing favorable conditions for natamycin synthesis [35]. Arginine regulates secondary metabolite synthesis through multiple mechanisms. For example, its metabolic products, such as nitric oxide and polyamines, can promote biofilm formation and cell growth and enhance stress resilience [36]. Additionally, arginine influences lipid metabolism, which facilitates the synthesis of specific secondary metabolites [37]. In AG-2, the accumulation of arginine may promote microbial growth and enhance stress resistance, thus supporting natamycin synthesis.

The final stage of L-isoleucine metabolism involves the cleavage of 2-methylacetoacetyl-CoA, producing acetyl-CoA and propionyl-CoA. Propionyl-CoA is then converted into methylmalonyl-CoA by a biotin-dependent carboxylase [38]. Through the branched-chain amino acid degradation pathway, isoleucine also contributes to methylmalonyl-CoA production. Chen et al. demonstrated that supplementing isoleucine during fermentation enhanced the supply of methylmalonyl-CoA, a key precursor of Tacrolimus, significantly increasing its yield in *Streptomyces tsukubaensis* [39]. Similarly, the elevated isoleucine concentration observed in AG-2 may play a crucial role in enhancing natamycin production by increasing the availability of acetyl-CoA and methylmalonyl-CoA. Valine can be converted into methylmalonyl-CoA through the branched-chain amino acid degradation pathway [40]. Supplementation experiments indicated that limited intracellular valine supply in the early fermentation phase (24 h) of AG-2 was one of the factors restricting natamycin production. However, valine supplementation at 36 h and 48 h did not significantly improve production, possibly because the elevated isoleucine levels in AG-2 compensated for the methylmalonyl-CoA deficiency. Intracellular valine levels in AG-2 surpassed those of the control strain at 96 h, suggesting that the initial valine limitation was alleviated. Excessive valine supplementation during the late fermentation phase, however, likely caused an oversupply, which unexpectedly inhibited natamycin synthesis.

Our results suggest that the extensive synthesis of natamycin in AG-2 may lead to excessive consumption of glutamate, which is compensated by the upregulation of nitrogen metabolism-related pathways. Glutamate and glutamine are key sources of amino groups in cellular metabolism, playing crucial roles in the synthesis of amino acids and other nitrogenous compounds [41]. Glutamine can be deaminated by glutaminase, removing its amide group to produce free ammonia and glutamate. The free ammonia participates in the synthesis of metabolites such as purines and pyrimidines, promoting cell growth and thereby enhancing natamycin production. Glutamate, through transamination reactions, provides amino groups for the synthesis of various intracellular amino acids (e.g., L-valine, L-leucine, and L-lysine). Therefore, enhanced nitrogen metabolism not only promotes cell growth but also increases the supply of amino acids related to precursor synthesis, significantly improving the natamycin production in AG-2.

## 5. Conclusions

This study proposes an innovative approach that significantly enhances natamycin production in *S. gilvosporeus* ATCC 13326 by combining optimized mutagenesis strategies with nitrogen metabolism regulation. The findings demonstrate that ARTP mutagenesis, coupled with 2-DG tolerance screening, effectively improved strain performance and markedly enhanced natamycin biosynthesis. The improved glycolysis and PP pathways, weakened TCA cycle, and upregulation of key genes in the natamycin biosynthetic cluster of AG-2 collectively enhanced the precursor supply and facilitated natamycin biosynthesis. Additionally, increased intracellular accumulation of amino acids such as cysteine, arginine, and isoleucine, along with strengthened nitrogen metabolism, contributed to improved natamycin biosynthesis and cell growth. The overexpression of GlnR further optimized nitrogen metabolism, increasing natamycin production to levels viable for industrial applications. This is the first attempt to enhance natamycin production by combining mutagenesis with nitrogen metabolism regulation, providing valuable insights into the efficient synthesis of natamycin and other polyene antibiotics. However, the regulatory mechanisms underlying natamycin biosynthesis remain unclear. In the future, physiological analysis and omics technologies can be utilized to uncover key regulatory pathways and elements, further enhancing natamycin production.

## Figures and Tables

**Table 1:** Evaluation of sixteen strain improvement strategies for natamycin production by *S. gilvosporeus*.

Strategy	Positive Mutation Rate	Production	Increase Multiple
UV and KH_2_PO_4_	8%	0.96 g/L	12.94%
UV and LiCl	2%	0.86 g/L	1.18%
UV and 2-DG	5%	0.93 g/L	9.41%
UV and Streptomycin	4%	0.88 g/L	3.53%
NTG and KH_2_PO_4_	12%	0.94 g/L	10.59%
NTG and LiCl	1%	0.87 g/L	2.35%
NTG and 2-DG	10%	1.17 g/L	37.65%
NTG and Streptomycin	1%	0.98 g/L	15.29%
DES and KH_2_PO_4_	16%	1.25 g/L	47.06%
DES and LiCl	14%	1.20 g/L	41.18%
DES and 2-DG	13%	1.21 g/L	42.35%
DES and Streptomycin	12%	1.06 g/L	24.71%
ARTP and KH_2_PO_4_	16%	1.31 g/L	54.12%
ARTP and LiCl	11%	1.27 g/L	49.41%
ARTP and 2-DG	17%	1.31 g/L	54.12%
ARTP and Streptomycin	7%	0.87 g/L	2.35%

## Data Availability

Data are contained within the article or Supplementary Materials.

## Supplementary Materials

The following supporting information can be downloaded at https://www.mdpi.com/article/10.3390/microorganisms13020390/s1: Figure S1: Lethality curves of *S. gilvosporeus* induced by UV mutagenesis (a), DES mutagenesis (b), ARTP mutagenesis (c), and NTG mutagenesis (d); Figure S2: Tolerance curves of *S. gilvosporeus* to 2-deoxyglucose (a), streptomycin (b), KH₂PO₄ (c), and LiCl (d); Figure S3: Effect of exogenous valine addition at 24, 36, 48, and 60 h on natamycin production of *S. gilvosporeus* AG-2. DCW: dry cell weight; Table S1: Primers for polymerase chain reaction employed in this study; Table S2: Transcriptional differences of key genes involved in natamycin biosynthesis clusters between AG-2 and ATCC 13326.
