# Supplementary material for: Improved Natamycin Production in Streptomyces gilvosporeus Through Mutagenesis and Enhanced Nitrogen Metabolism
Source: Microorganisms. 2025 Feb 10;13(2):390. doi: 10.3390/microorganisms13020390 (PMC11857858; doi:10.3390/microorganisms13020390)
Supplement: Supplementary file 1 [file microorganisms-13-00390-s001.zip › microorganisms-3376521-supplementary.pdf]

Fig. S1

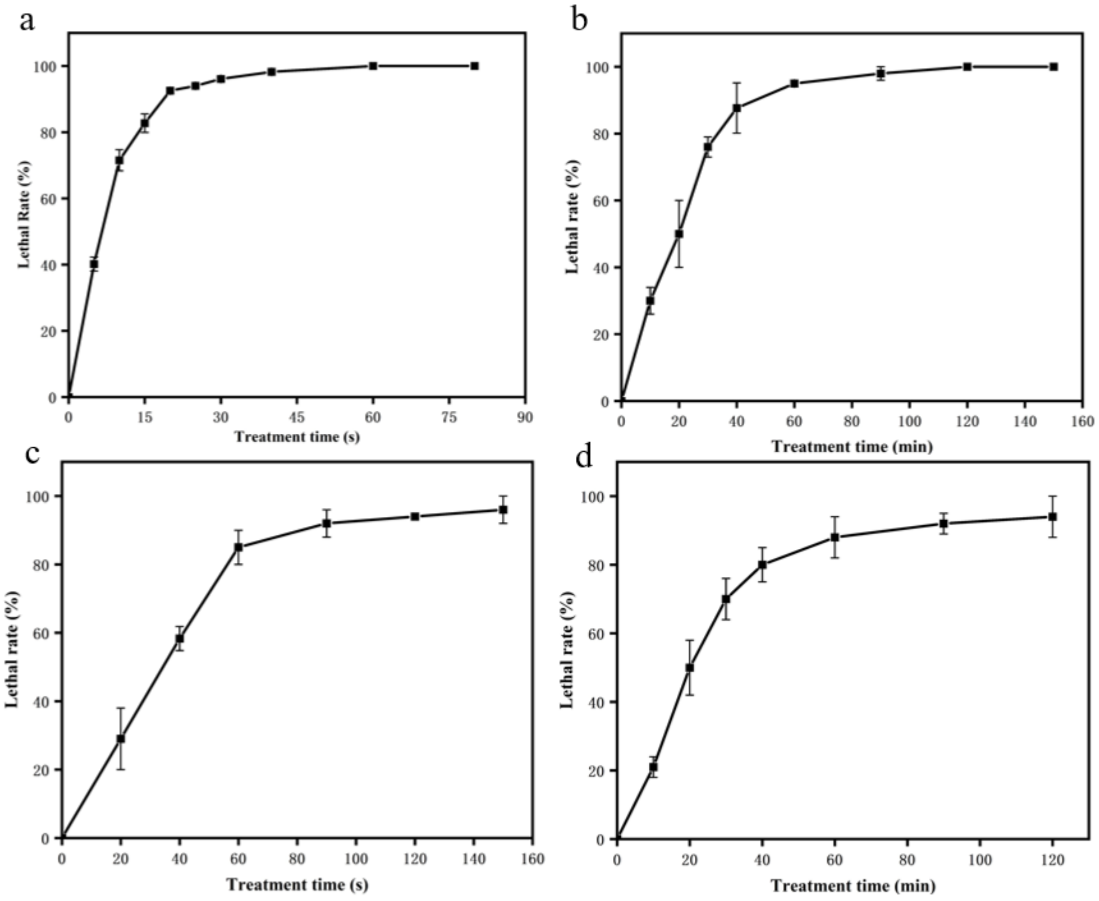

Fig. S1 Lethality curves of *S. gilvosporeus* induced by UV mutagenesis (a), DES mutagenesis (b), ARTP mutagenesis (c), and NTG mutagenesis (d)

Fig. S2

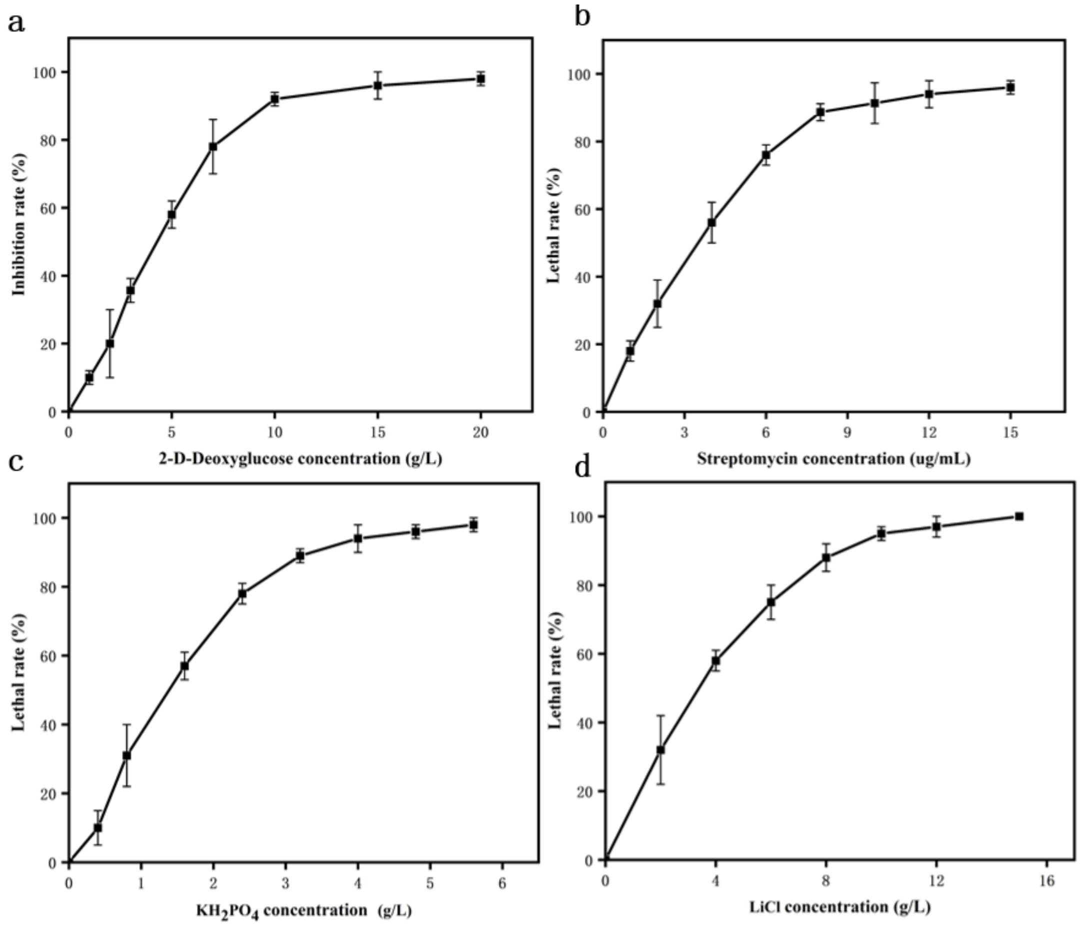

Fig. S2 Tolerance curves of *S. gilvosporeus* to 2-deoxyglucose (a), streptomycin (b), KH_2PO_4 (c), and LiCl (d)

Fig. S3

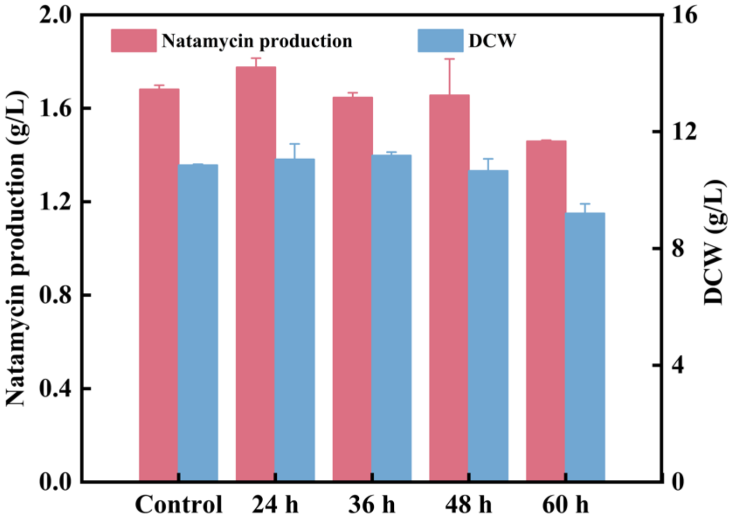

Fig. S3 Effect of exogenous valine addition at 24, 36, 48, and 60 h on natamycin production of *S. gilvosporeus* AG-2. DCW：dry cell weight.

Table S1 Primers for polymerase chain reaction employed in this study

Gene name	Primer	Primer sequence (5'-3')
sgnS0	sgnS0-F	ACACCTCGTCCGTCAACTC
	sgnS0-R	TCGACGACACCACCTACAC
sgnS1	sgnS1-F	TCGTGATCGTCGGCATGAG
	sgnS1-R	CAGGTCGTCTCCAGCAACA
sgnS2	sgnS2-F	CGCTACGCAGAGGTCTTCA
	sgnS2-R	CGAAGCCGAGGTCACTGAA
sgnS3	sgnS3-F	GGTCGGTCAAGTCCAACCT
	sgnS3-R	GCCTGCTCCACGATCACAT
sgnS4	ssgnS4-F	CAAGTCCAACCTCGGTCACA
	sgnS4-R	TGGCTGCTCAAGGATGATGT
sgnC	sgnC-F	GTTGGCGAACAGCGAGTAG
	sgnC-R	CTGCGGAGACGACCTCAATA
sgnG	sgnG-F	TCGAAGGCGTTCTCGTCAT
	sgnG-R	TGCTGATGCTCCTGCTCAT
sgnK	sgnK-F	CGAAGGTCCGCAGCTTGAA
	sgnK-R	GAAGTTCCGCCGTCTCCAA
sgnI	sgnI-F	GGATCGTCGCCGAACTGAA
	sgnI-R	GGAGCTGAGGAAGAAGTGACC
sgnJ	sgnJ-F	TGGTGTCCGAACTGTCCTT
	sgnJ-R	CGACCTTGCCGAACATCTC
sgnE	sgnE-F	AACAGGTGACGGCAGGAGA
	sgnE-R	CGAGGTCATCTACGGCAACA
sgnF	sgnF-F	TCGTCGTCCTGGTCGAAGAG
	sgnF-R	ATGCGTATCACCGTTGACCC
sgnL	sgnL-F	CAGATTGTTGGTGCGGTACAG
	sgnL-R	CGTTCACCGTGTCCTGGAA
sgnD	sgnD-F	GGTGAACAGCTTGGTGAACATC
	sgnD-R	GCCAGCGAGGACATGGAATT
sgnA	sgnA-F	TCACCATCACCACCGACAT
	sgnA-R	GCCGCACCAATTCCGAAT
sgnB	sgnB-F	CGTTGTGCTCGTCGAAGAC
	sgnB-R	CGTTCTCCGTGCTGACCAT
sgnT	sgnT-F	GGTGTCCACGGAGAGCATT
	sgnT-R	CGCCGAGGTAGACGAGATAC
sgnH	sgnH-F	TCCTCAATGCCGCCTCGTA
	sgnH-R	GGAGTGGAAGACCGTGATCG
sgnM	sgnM-F	GGCTCGTCCGAGGACATAT
	sgnM-R	GAGGATCTTCTTGCGTTTGGT
sgnR	sgnR-F	CCAGCATCGCCTTCCAGTA
	sgnR-R	CCGCTCCTACAACACCTTCA
nasA	nasA-F	GCTGACCTCTCCTCTGGTAC

	nasA-R	GCTGACCTCTCCTCTGGTAC
nasB	nasB-F	CCTACAACCGTGTGCTGCT
	nasB-R	TGGCGTTCCATCAGGTGCT
gdhA	gdhA-F	TGATGCGGTTCTGCCAGTC
	gdhA-R	CGACCTCCTTGATCTGCTTGA
nirB	nirB-F	CCTGGTCAACGACGAACTG
	nirB-R	ACCGAATACGAGCCATTGC
glnR	glnR-F	GGACCTGACCTTCAAGGAGTT
	glnR-R	ACCGCCGAAGTAGTCATAGC
glnA	glnA-F	GCATCAACGCCGAGGTCAT
	glnA-R	CAGCAGGTGTCCACGATCA
gltD	gltD-F	CACATCAACCGCCGTATCG
	gltD-R	GGAGGTGACCTTGTAGAGCAT

Table S2 Transcriptional differences of key genes involved in natamycin biosynthesis clusters between AG-2 and ATCC 13326.

Gene	Description	Fold Change
sgnS0	Type I polyketide synthase	8.45
sgnS1	Type I polyketide synthase	3.71
sgnS2	Type I polyketide synthase	5.83
sgnS3	Type I polyketide synthase	4.64
sgnS4	Type I polyketide synthase	7.37
sgnA	ABC transporter ATP-binding protein	13.76
sgnB	ABC transporter ATP-binding protein	28.81
sgnC	DegT/DnrJ/EryC1/StrS family aminotransferase	19.43
sgnD	Cytochrome P450	19.53
sgnE	GMC oxidoreductase	7.71
sgnF	Ferredoxin	11.89
sgnG	Cytochrome P450	27.86
sgnI	Alpha/beta fold hydrolase	16.12
sgnJ	GDP-mannose 4,6-dehydratase	26.71
sgnK	Glycosyltransferase	23.92
sgnL	Tyrosine-protein phosphatase	-1.65
sgnM	LuxR C-terminal-related transcriptional regulator	9.30
sgnR	AfsR/SARP family transcriptional regulator	-1.22
sgnH	MFS transporter	2.45
